# Application of a Wireless and Contactless Ultrasonic System to Evaluate Optimal Sawcut Time for Concrete Pavements

**DOI:** 10.3390/s22187030

**Published:** 2022-09-16

**Authors:** Homin Song, Jinyoung Hong, Young-Geun Yoon, Hajin Choi, Taekeun Oh

**Affiliations:** 1 Department of Civil and Environmental Engineering, Gachon University, Seongnam-si 13120, Korea; 2 School of Architecture, Soongsil University, Seoul 06978, Korea; 3 Department of Safety Engineering, Incheon National University, Incheon 22012, Korea

**Keywords:** wireless, contactless, ultrasonics, saw-cutting time, concrete, pavement

## Abstract

A recently developed contactless ultrasonic testing scheme is applied to define the optimal saw-cutting time for concrete pavement. The ultrasonic system is improved using wireless data transfer for field applications, and the signal processing and data analysis are proposed to evaluate the modulus of elasticity of early-age concrete. Numerical simulation of leaky Rayleigh wave in joint-half space including air and concrete is performed to demonstrate the proposed data analysis procedure. The hardware and algorithms developed for the ultrasonic system are experimentally validated with a comparison of saw-cutting procedures. In addition, conventional methods for the characterization of early-age concrete, including pin penetration and maturity methods, are applied. The results demonstrated that the developed wireless system presents identical results to the wired system, and the initiation time of leaky Rayleigh wave possibly represents 5% of raveling damage compared to the optimal saw cutting. Further data analysis implies that saw-cutting would be optimally performed at approximately 11.5 GPa elastic modulus of concrete obtained by the wireless and contactless ultrasonic system.

## 1. Introduction

Concrete is one of the most popular materials in civil engineering. Along with the increasing need for structural inspection, ultrasonic methods have been employed to evaluate the quality of materials in situ. Ultrasound is beneficial for characterizing the soundness of materials because the wave parameters such as velocity and amplitude are directly related to the mechanical properties of the materials [1]. However, the contact-based methods have limited application due to the coupling procedure [2]. First, the significant acoustic impedance mismatch between concrete and ultrasonic transducers made by piezoelectric materials requires a gel-type couplant, which is not practical in the field. Second, the surface roughness of concrete frequently causes irregular contact of the transducers. To avoid the multiple scattering effects by coarse aggregate, a low-frequency transducer below 100 kHz is typically used for standard concrete inspection applications [3]. Moreover, the piezoelectric transducers usually have relatively large dimensions of the contact surface.

Recent advances in non-contact ultrasonic sensing technology have broadened the scope of nondestructive inspection of various engineering materials including concrete [4]. Zhu et al., (2004) demonstrated that the leakage portion of surface waves measured by contactless sensors provides information on internal damage in concrete [5]. Several studies have proved the effectiveness of the contactless sensing technology to inspect concrete structures [6]. Neuenschwander et al., (2006) showed the measurement of leaky Rayleigh waves from mortar samples using a fully contactless ultrasonic system [7]. Further developments in the contactless ultrasonic system field have been reported [8,9,10], and unique signal processing algorithms have also been suggested in addition to the contactless ultrasonic hardware system [11,12,13,14]. As described in previous studies, the contactless ultrasonic system is an efficient method for the inspection of concrete structures, overcoming the drawbacks of the conventional contact-based approach.

On top of inspection applications (e.g., damage detection), the contactless ultrasonic system enables the provision of practical information on early-age material characteristics, such as the setting time. Conventional methods to determine a setting time are based on pin penetration tests, where a physical pressure load is applied to the surface of a concrete specimen. However, the methods recommend wet sieving to filter out coarse aggregate such as gravel, which is a significantly labor-intensive and time-consuming procedure. Choi et al., (2016) first demonstrated that the initiation of leaky Rayleigh waves is a useful tool to determine the final setting of early-age cementitious materials without physical contact with the specimens [12]. Moreover, the incident angle of ultrasonic waves is a key parameter in defining the degree of the setting, where the larger angles provide the earlier Rayleigh wave initiation, resulting in a faster definition of the setting time. Hong et al., (2020) evaluated the effectiveness of contactless ultrasonic measurements in monitoring the stiffening behavior of concrete along with the maturity method [15]. Hong and Choi (2021) further developed a contactless ultrasonic measurement hardware system and suggested a signal processing algorithm to investigate the elastic modulus of early-age cementitious materials [16].

In the characterization of early-age concrete, the contactless ultra-sonic system showed great potential for field applications. Tran and Roesler (2020) applied the contactless ultrasonic system to the saw-cutting initiation time in concrete pavements and suggested the framework to predict the relationship between the saw-cut initiation time and final setting time [17]. Saw cutting is required to control the quality of jointed plain concrete pavements to prevent early-age shrinkage cracks. Therefore, the contraction joints are constructed at the surface of the pavements using the saw cut. The saw cutting is a highly time-dependent procedure that causes raveling damage at a too early stage of concrete hardening or random cracking at a too late stage of concrete hardening. A proper saw-cut time window has been investigated based on the levels of produced damage using computer vision [18,19]. Tran and Roesler (2021) further evaluated the incident angle effect of the contactless ultrasonic system to identify the optimal setting definition for the saw cutting [20]. Based on the previous studies, the delayed initiation time of leaky Rayleigh wave compared to the final setting is required to define the optimal saw-cutting time. Therefore, the smaller incident angle will give a better correlation between the optimal saw-cutting time and the initiation of leaky Rayleigh waves.

In this study, the optimal saw-cutting time is investigated using the concept of a contactless ultrasonic scheme. The uniqueness of this study over previous research lies in (1) the improvement of the sensor network of the contactless system using wireless data transfer for possible field applications, and (2) the development of a signal processing and data analysis scheme to determine the optimal saw-cutting time. Moreover, we optimized the critical testing parameters of the contactless ultrasonic system for application to saw-cutting time, such as the incident angle, measurement time intervals, and the number of sensor array elements. The theoretical background of leaky Rayleigh wave propagation in early-age concrete is supported using numerical simulations. Moreover, the developed hardware and signal processing algorithm were experimentally validated and compared to the conventional methods, such as the pin penetration and maturity methods.

## 2. Methodology

### 2.1. Theoretical Background of Leaky Rayleigh Wave Propagation in Early-Age Concrete

Rayleigh wave is one of the surface waves, propagating at the surface of a medium. The wave motion is elliptical rotation, which is caused by the surface guidance of body waves. The portions of longitudinal and shear waves generate the unique behavior of wave propagation. The relationship between material properties and Rayleigh wave velocity is given by

(1) $$V_{R}=\frac{0.87+1.12\nu }{1+\nu }\sqrt{\frac{E}{2\rho \left(1+\nu \right)},}$$

where E is the elastic modulus, ρ is the mass density, and ν is Poisson’s ratio [21].

At the joint-half space of air and concrete, small portions of the Rayleigh wave en0.ergy are leaked into the air (called leaky Rayleigh waves). A contactless ultrasonic measurement system measures the leaky Rayleigh wave without a physical coupling procedure to the surface of a concrete element. In non-destructive evaluation, the measurement of leaky Rayleigh waves has the great benefit of non-invasive material characterization.

Concrete is the composite material consisting of cement, and fine and coarse aggregate. The early-age concrete mixture is in a liquid state when water is added, and the hydration reaction makes the mixture harden in time. Conventionally, the degree of hardening at the initial stage is defined by the initial and final setting, generally representing the initiation of chemical adhesion and the physical stiffening of the mixture, respectively. The solid-state of concrete is considered as the time after the final setting. Leaky Rayleigh waves are measurable when the medium enables stress transfer, implying the propagation of both longitudinal and shear waves inside the medium. Therefore, the initiation of leaky Rayleigh waves represents the critical phase of shear resistance development in the medium, which is more relevant to the definition of the final setting.

The stiffening scenario with respect to leaky Rayleigh wave propagation is numerically demonstrated using the finite element method. The commercially available software, COMSOL Multiphysics, was used to analyze the propagation of the leaky Rayleigh wave at the joint-half space. The Multiphysics model included the air and concrete, where the ultrasonic excitation and sensing locations were positioned in the air. The details of the numerical simulation are described in Table 1. Note that both materials were assumed to be homogeneous with respect to the applied wavelength, where the random scattering by aggregate was not considered. In addition, acoustic absorbing layers were placed around the model to eliminate reflection by the boundary, simulating infinite spaces. Therefore, the generated ultrasound was only guided by the joint between the air and concrete, implying the surface of the concrete. To investigate the stiffening phenomenon in concrete, a series of numerical simulations were carried out, where 26 cases of the elastic modulus of concrete ranging from 0.1 GPa to 25 GPa were considered in 1 GPa increments.

**Figure 1:**
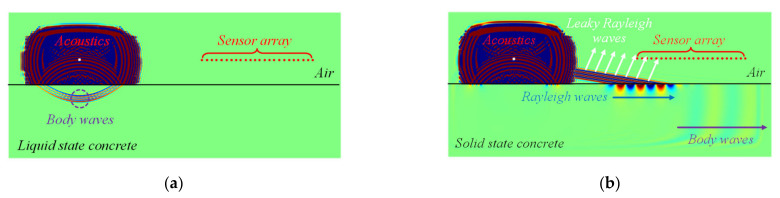
Wavefield snapshots generated from numerical simulations: ultrasonic wavefields at t = 300 µs for the cases of (**a**) beginning of hydration (*E* = 0.1 GPa) and (**b**) fully hydrated concrete (*E* = 25 GPa).

The simulation results presenting wavefield snapshots are shown in Figure 1. The presented wavefield images were captured 300 microseconds after the ultrasonic excitation. The ultrasonic excitation was placed 50 mm apart from the surface of concrete acting as a point source shown on the left side of the images. At the beginning of hydration (*E* = 0.1 GPa) shown in Figure 1a, Rayleigh waves are not generated while others such as acoustic and body waves are identified in the air and concrete, respectively. After the modulus is developed to a certain amount (e.g., *E* = 1 GPa), Rayleigh waves propagate at the surface of concrete, and the corresponding leaky waves are observed in the air. In the hardened concrete case (*E* = 25 GPa) seen in Figure 1b, the body waves exhibit a faster propagation speed and a larger wavelength compared to those in fresh concrete seen in Figure 1a. Also, the velocity of Rayleigh waves is much faster than that of the acoustics (Figure 1b). Therefore, the contactless sensors receive the leaky Rayleigh waves first and direct acoustic waves later. Note that the amplitude of leaky Rayleigh waves is intrinsically lower than that of the acoustics. As the elastic modulus in concrete is developed, leaky Rayleigh waves propagate faster while the acoustic wave speed remains constant.

**Figure 2:**
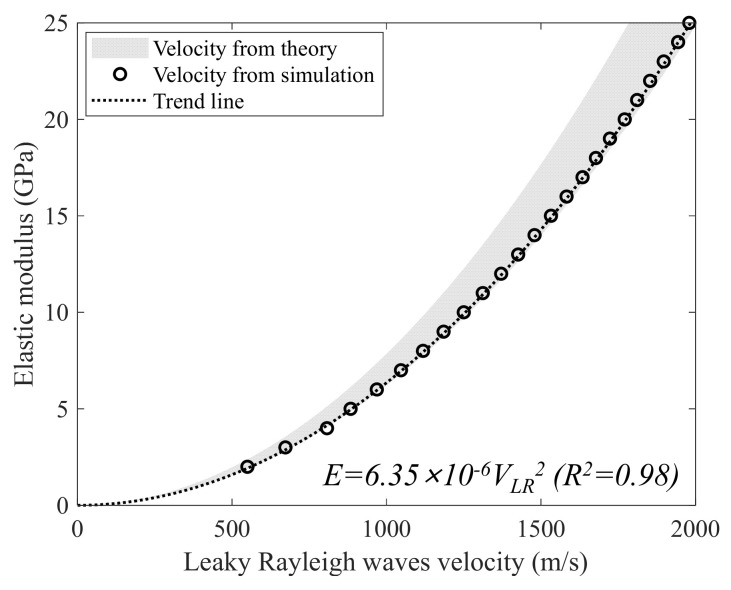
Derived relationship between the leaky Rayleigh wave velocity ($V_{LR})$ and elastic modulus (*E*). Note that the range of theoretical Rayleigh wave velocity was calculated across the range of Poisson’s ratio (0.15~0.3) and mass density (2200~2600 kg/m^3^).

Based on the results of numerical simulations, the direct relationship between the leaky Rayleigh wave velocity and elastic modulus of concrete was derived as shown in Figure 2. The velocity of leaky Rayleigh waves at each state of concrete was simply calculated from signals received by sensor arrays. The obtained relationship was presented as the dotted line in Figure 2 while the theoretical range of Rayleigh waves based on Equation (1) is indicated in the shaded area. An equation (see Figure 2) was derived from the trend line to estimate the elastic modulus directly from the measured leaky Rayleigh wave velocity. This simple equation is useful in practice, considering that information on Poisson’s ratio and mass density are typically lacking on construction sites. The suggested equation conservatively correlates the leaky Rayleigh wave velocity and the modulus of elasticity, which would be a good estimation of the elastic modulus of concrete in the field.

### 2.2. Sensor Network for Wireless Data Transfer and Signal Processing

**Figure 3:**
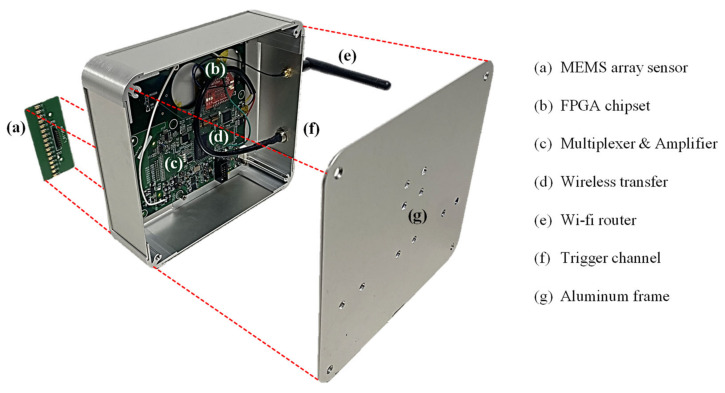
The developed wireless and contactless ultrasonic measurement system.

To measure the leaky Rayleigh wave responses from concrete pavements, a wireless and contactless ultrasonic system was developed. The system includes two printed circuit boards (PCBs) encapsulated by an aluminum frame. Each PCB is designed for sensing and data acquisition parts, respectively. The sensing board consists of 16 MEMS (Microelectromechanical systems) ultrasonic sensor elements placed with 5 mm spacing. The data acquisition board is composed of several components, including a field-programmable gate array (FPGA), multiplexer, amplifier, and Wi-Fi module. The details of the wireless system are presented in Figure 3.

The acoustic pressure is measured using the MEMS sensor array, and the collected analog signals are amplified 2000 times at the center frequency of 50 kHz. Then, the analog signals are converted to digital signals with a sampling rate of 2 MS/s and a dynamic resolution of 14 bits. The data acquisition process is repeated 16 times through each MEMS sensor element of the array, and the acquired data are wirelessly transferred to a personal computer. The stored data can be checked in real-time with programmed software, and measurement parameters such as the number of time-averaging can be set by an operator. The developed system is significantly improved in terms of the overall structure of the sensor network compared to the previously applied wired unit [16]. First, wireless data transferring has great potential for field applications. The Internet of Things broadens the range of wireless data acquisition. The signal strength was evaluated as good (−50 to −60 dBm) with a maximum distance of 5 m [22]. Second, the system is operated by a rechargeable battery, which is practical in pavement construction sites where electric power is frequently unavailable. Third, the number of sensors in the wireless system is twice that of the wired system, which increases the wavenumber resolution for data analysis in the frequency–wavenumber domain. With 16 MEMS sensors placed with 5 mm spacing, the length of the array is 80 mm, which is longer than the wavelength of Rayleigh waves (assuming 50 mm at 50 kHz) in hardened concrete.

**Figure 4:**
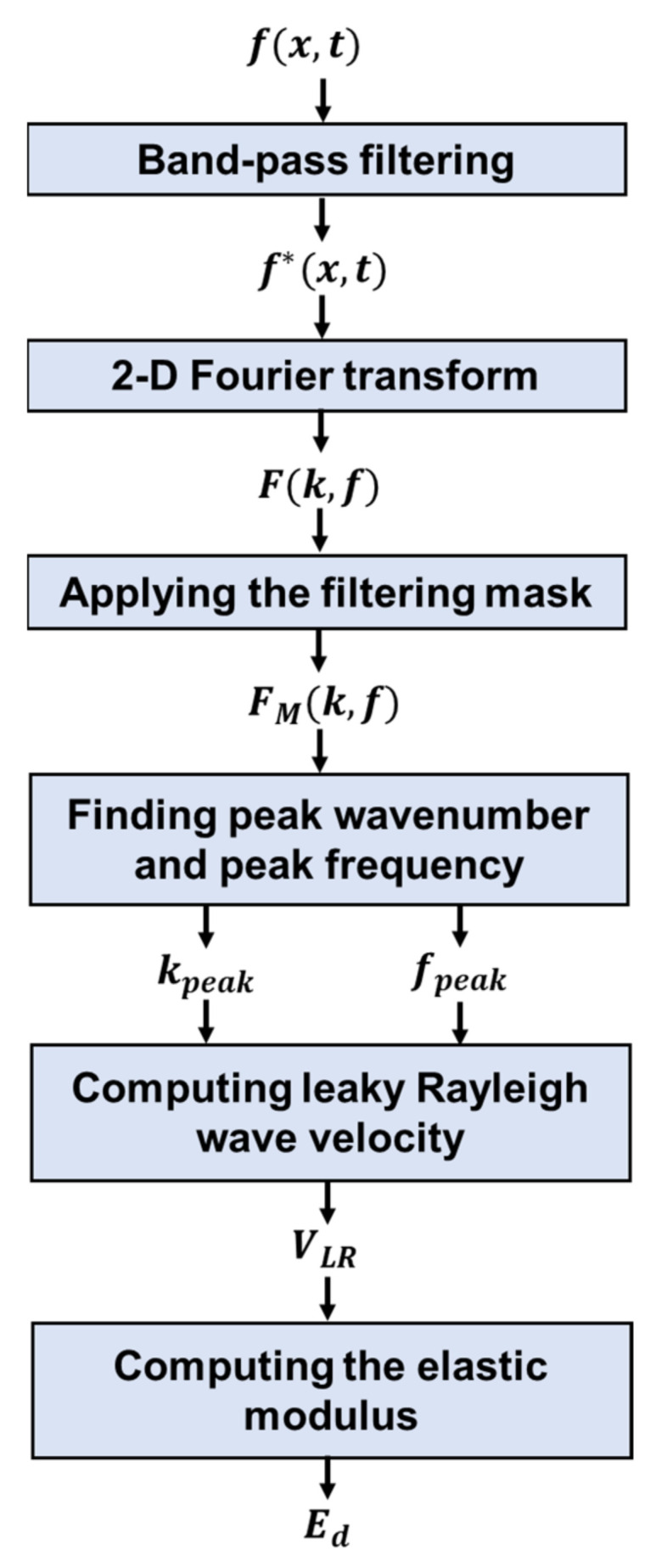
A flowchart of the proposed signal processing scheme.

In addition to the hardware development, we propose a signal processing approach to computing the leaky Rayleigh wave velocity from measured signals. An overview of the proposed signal processing approach is illustrated in Figure 4.

First, bandpass filtering is applied to the measured signal set $f\left(x,t\right)$ to suppress noises other than the frequency band of excitation (40 to 50 kHz). Then, a two-dimensional (2-D) Fourier transform is applied to the bandpass filtered signal set $f^{*}\left(x,t\right)$ to convert the signal set from time–space (*t-x*) to frequency–wavenumber (*f-k*) domains, given by

(2) $$F\left(k,f\right)=\int_{-\infty }^{\infty }\int_{-\infty }^{\infty }f^{*}\left(x,t\right)e^{-j2\pi \left(ft+kx\right)}dxdt,$$

where $F\left(k,f\right)$ is the converted *f-k* domain signal set, and *j* is the imaginary number. Next, a region of interest in the *f-k* domain is highlighted by multiplying a filtering mask $M\left(k,f\right)$ and $F\left(k,f\right)$, given by

(3) $$F_{M}\left(k,f\right)=F\left(k,f\right)M\left(k,f\right),$$

where the filtering mask $M\left(k,f\right)$ is defined as

(4) $$M\left(k,f\right)=\left\{\begin{array}{cc} 1 & \mathrm{if}\;830\;m/s\leq \frac{f}{k}\leq 3100\;m/s\\ 0 & otherwise. \end{array}\right.$$

Note that the filtering operation shown in Equation (3) maintains wave components with the Rayleigh wave velocity of typical concrete while suppressing other unwanted components including noise. Then, peak frequency ($f_{peak}$) and wavenumber ($k_{peak}$) are obtained by picking *f* and *k* values at the maximum amplitude of $F_{M}\left(k,f\right)$:

(5) $$arg\;\underset{k,f}{max}\;\left|F_{M}\left(k,f\right)\right|$$

Finally, Leaky Rayleigh wave velocity ($V_{LR}$) is computed as

(6) $$V_{LR}=\frac{f_{peak}}{k_{peak}}$$

Once $V_{LR}$ is obtained, the modulus of elasticity can be computed, given by

(7) $$E=6.35\times 10^{-6}V_{LR}{}^{2}$$

Note that Equation (7) was derived from the simulation results shown in Figure 2. Monitoring the development of $V_{LR}$ or *E* over time, the hardening process of early-age concrete can be monitored. Also, the optimal saw-cut timing can be determined using the $V_{LR}$ or *E* data, which is presented in Section 3.

### 2.3. Conventional Methods: Maturity and Pin Penetration

To characterize the state of early-age concrete, conventional methods such as maturity and pin penetration methods were also performed. The pin penetration method (ASTM C403) is a standard test method to determine the setting time of concrete mixtures [23]. In this test method, pins having a different support area are used to measure the penetration resistance of the sieved mortar fraction from a concrete mixture, across different hardening stages of concrete. Dividing the applied load with the area of the used pin, a penetration resistance value is computed. Using the measured penetration resistance values, a smooth trend curve can be obtained, which visualizes the hardening of the tested concrete mixture. The initial and final setting times are determined by picking the time points that correspond to 3.5 MPa and 27.6 MPa, respectively.

Despite its wide acceptance as a means to determine the setting time of concrete, the pin penetration method has several limitations. First, performing the pin penetration test is highly labor-intensive. As the pin penetration method is not applicable directly to concrete with coarse aggregate, wet sieving needs to be carried out to extract the mortar fraction of the concrete mixture. Also, the test results can vary depending on the proficiency of the operator. To obtain reliable setting times, the operator needs to be trained for the test procedure. Moreover, the pin penetration test is typically carried out in a controlled laboratory environment that can be different from that of an actual construction site (e.g., temperature and relative humidity).

The maturity method (ASTM C1074) is a standard practice to estimate concrete strength using measured temperature data [24]. Two maturity functions are widely used to compute the maturity index from the measured temperature history: (a) Nurse–Saul (NS) and (b) Freiesleben–Hasen and Pedersen (FHP) methods. The NS method is used to compute the temperature–time factor, and the FHP method to compute the equivalent age of concrete mixtures. In this study, the FHP method is used to compute the equivalent age given by

(8) $$t_{e}=\sum e^{-Q\left(\frac{1}{T_{a}}-\frac{1}{T_{s}}\right)}\Delta t$$

where $t_{e}$ is the equivalent age at a specified temperature $T_{s}$, *Q* the activation energy divided by the ideal gas constant, and $T_{a}$ the average temperature of concrete during the time interval Δt. Typically, a calibration curve that relates between $t_{e}$ and the compressive strength is constructed in a laboratory setting. Then, the calibration curve constructed for a given mixture design is used to predict the compressive strength of early-age concrete on site using the measured temperature data. In this study, we use the equivalent age $t_{e}$ as an auxiliary index to help determine the optimal saw-cutting time, rather than to predict the compressive strength of concrete mixtures.

Like the pin penetration test, the maturity method has its own limitations. First, the laboratory curing temperature may not represent that of the concrete element on the actual construction site. Also, the strength of concrete cannot be estimated directly from the temperature measurement data; a calibration curve needs to be constructed before actual strength estimation is carried out, which is time-consuming. The maturity method does not capture local strength variations within a concrete element.

## 3. Experimental Validation

### 3.1. Concrete Specimen and Test Procedures

**Figure 5:**
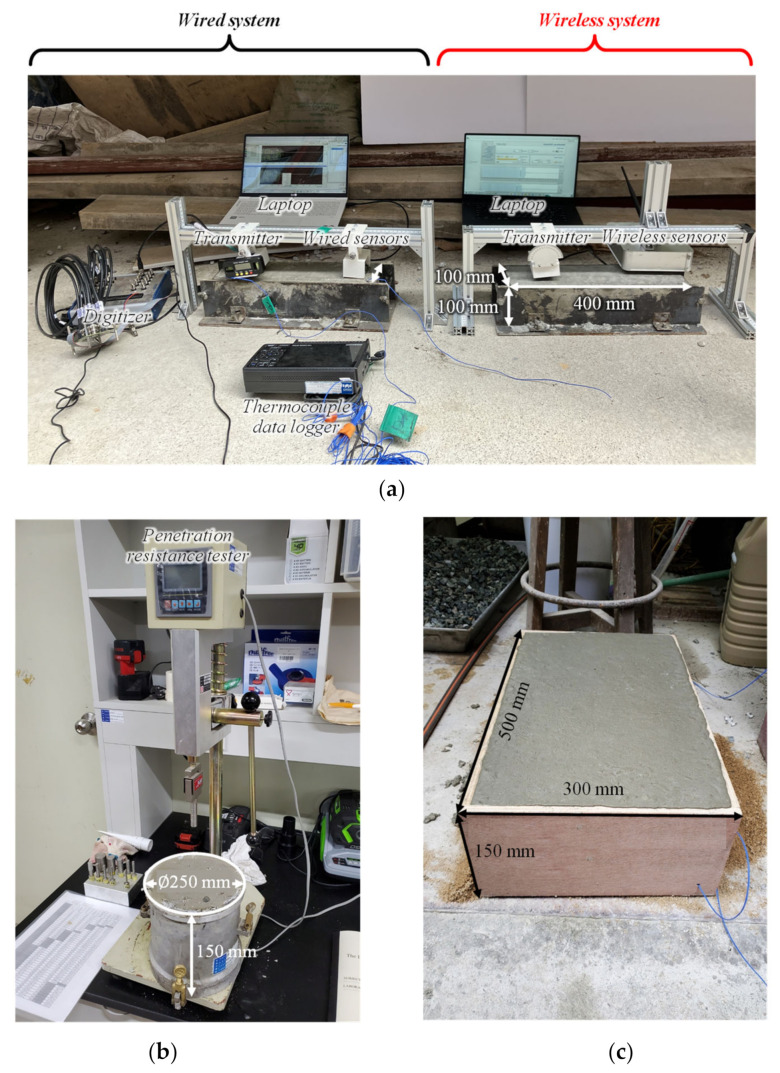
Test setups: (**a**) contactless ultrasonic systems (wired and wireless systems) with thermal gauges, (**b**) pin penetration test setup, and (**c**) a slab specimen for saw cuts.

The developed wireless and contactless ultrasonic hardware system and proposed signal processing approach were validated through experiments. All the early-age concrete specimens used in this study were produced with the mixture design proportions summarized in Table 2. In addition to the mixture proportions, several early-age properties including slump and the volume of entrained air are also seen in the table. The presented mixture design proportions are widely used to construct concrete pavements in South Korea. From a concrete batch produced with the mixture proportions, four concrete specimens were prepared which are shown in Figure 5. Two beam specimens with dimensions of 400 mm × 100 mm × 100 mm were used for contactless ultrasonic monitoring of concrete hardening (Figure 5a): one used for testing the developed wireless and contactless ultrasonic system and the other for testing the wired ultrasonic system. Thermal gauges were inserted in the beam specimens to monitor the temperature history for the maturity method. A cylindrical specimen with a diameter of 250 mm and a height of 150 mm shown in Figure 5b was used for the pin penetration test. A slab specimen (500 mm × 300 mm × 150 mm) seen in Figure 5c was prepared to apply saw cuts at different time instances.

Once the specimens were prepared, contactless ultrasonic tests and maturity tests were carried out. For the contactless ultrasonic measurement tests (both the wireless and wired systems), a contactless air-coupled transmitter (PID-615089, SensComp) with a center frequency of 48 kHz generated ultrasonic Rayleigh waves in the tested specimens, and the corresponding leaky Rayleigh wave responses were measured using an array of MEMS microphones. Measured analog signals were digitized at a sampling rate of 2 MS/s using a data acquisition device (USB 6366, National Instruments). An incident angle was set to 5° for the air-coupled transmitter. To improve the signal-to-noise level, 50 times time averaging was applied. Leaky Rayleigh wave responses were automatically measured every minute and stored in laptop computers.

Pin penetration tests were carried out following ASTM C403. More details about the test method can be found in [23]. For the saw-cut test, saw cuts were introduced at different time instances from the start of hydration: 510, 558, 601, 645, 683, 710 and 1020 min. To evaluate the saw cut quality, optical images were collected from the top surface of the slab specimen after saw cuts were introduced.

### 3.2. Results from the Wireless and Contactless Ultrasonic System

**Figure 6:**
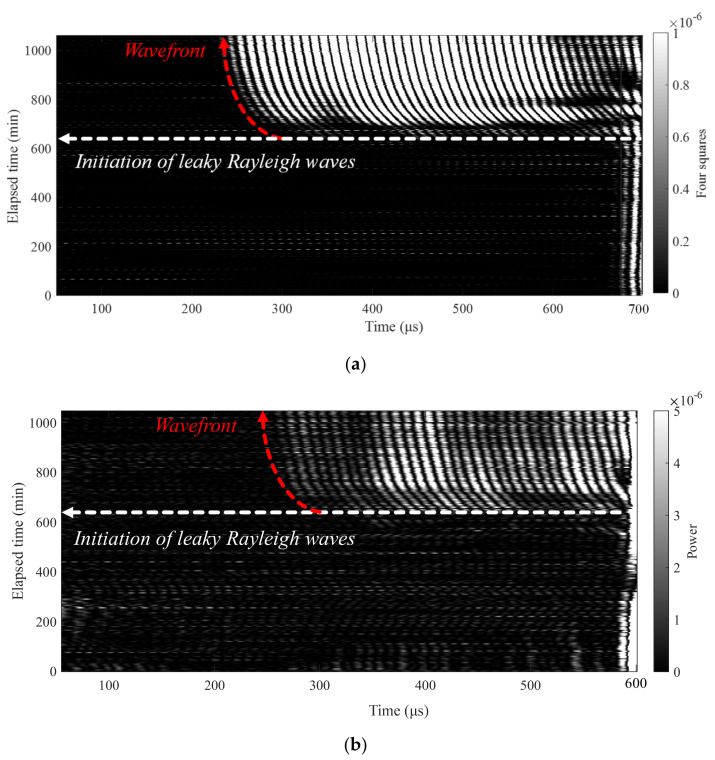
B-scan images obtained from the (**a**) wired system, and (**b**) wireless system.

Leaky Rayleigh wave signals measured using the contactless ultrasonic measurement systems are presented in Figure 6. To visualize the setting and hardening behavior of the early-age concrete specimens in a continuous time frame, all the time-domain signals collected during the monitoring period were stacked together, and the signal amplitudes are visualized in a grayscale colormap (hereafter, B-scan image). Figure 6a,b show the B-scan images collected using the wired and wireless ultrasonic measurement systems, respectively. For both B-scan images, the leaky Rayleigh wave initiation time and the wavefront development over the elapsed time are indicated. Compared to the wired measurement case (Figure 6a), the wireless measurement case (Figure 6b) shows an acceptable signal-to-noise level, revealing that the leaky Rayleigh wave initiation and the wavefront development can be clearly observed. The leaky Rayleigh wave initiation time, which represents the final setting time, obtained from the wireless measurement case (650 min) was identical to that obtained from the wired measurement case (650 min). Note that the wavefront increment shown in Figure 6 resulted from the development of leaky Rayleigh wave velocity due to the hardening of the tested concrete specimen. The B-scan images shown in Figure 6 demonstrate the high field applicability of the developed wireless and contactless ultrasonic system, enabling automated and continuous monitoring of concrete hardening without wired power transmissions and data transporting cables.

Despite the good agreement in the leaky Rayleigh wave initiation time and wave-front development, the wired and wireless measurement systems show differences in signal-to-noise level in the B-scan images (Figure 6). The signal-to-noise ratio (SNR) after the leaky Rayleigh wave initiation time is 20.9 dB and 11.3 dB for the wired and wireless measurement systems, respectively. We believe that different power supply systems might have caused the different signal-to-noise levels: a stable wired power supply for the wired system and a less stable battery power supply for the wireless system. Nevertheless, the lower SNR achieved by the wireless system does not affect the estimation of concrete setting time and continuous monitoring of hardening behavior.

### 3.3. Results from Conventional Methods

**Figure 7:**
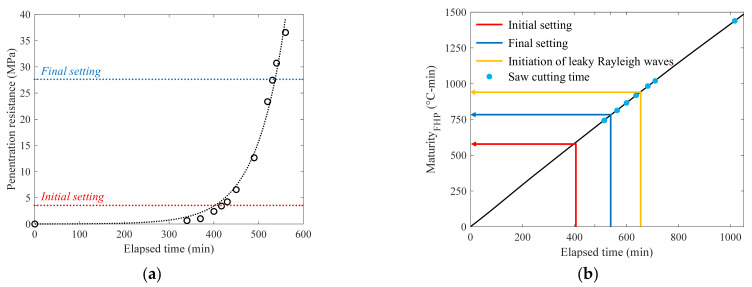
Test results from the (**a**) pin penetration test, and (**b**) maturity method.

The results from the conventional methods are presented in Figure 7. From the pin penetration test results seen in Figure 7a, the initial and final setting times of the mixture were identified as 401 and 538 min, respectively. Note that the setting time was defined based on the trend line as described in ASTM C403. It is confirmed that the setting time defined by leaky Rayleigh waves was approximately two hours later than the final setting time determined by the pin penetration test, which is different from a previous study [17]. This is because the 5-degree incident angle of ultrasound used in this study is for the critical angle of hardened concrete, which delayed the energy concentration at the surface of concrete. Correspondingly, the initiation of leaky Rayleigh waves is also delayed which would be appropriate for the assessment of saw-cut initiation.

The equivalent age of the mixture is shown in Figure 7b, including the setting times and operated saw-cut times. The six saw-cutting operations were carried out near the final setting time from 510 to 710 min, and an additional saw cut (seventh) was introduced at 1020 min as a reference. With respect to the initiation of leaky Rayleigh waves, the fifth and sixth saw cuts were performed after the setting time. The corresponding maturity of the concrete itself does not have physical meaning; however, the values could be used to the same mixture proportions of concrete for an approximate prediction of the setting time.

## 4. Discussion

### 4.1. Raveling Damage Identification

**Figure 8:**
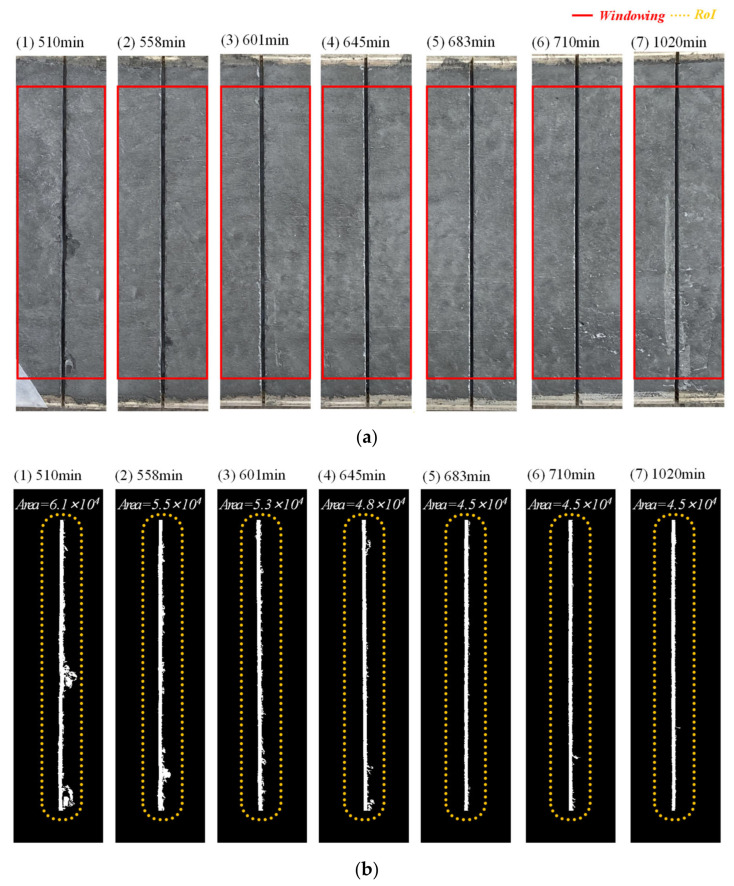
Saw cut damage identification: (**a**) saw cut images cropped from photos, and (**b**) the corresponding binarized images. Note that the red solid box indicates the windowed region, and the yellow box indicates the RoI.

Raveling damages caused by a saw cut that is too early were observed on the top surface of the slab specimen as shown in Figure 8a. To quantify the degree of raveling damage, image analysis was performed. Each saw-cut region was cropped from the photo taken from the slab specimen, and the cropped images were inversely binarized (Figure 8b), where white and black pixels represent cut and non-cut areas, respectively. Then, the area of the white pixels inside the region of interest (RoI) was calculated and defined as the saw-cut area (SA). The overall image processing was performed using MATLAB functions: the ‘im2bw’ function (with a luminance threshold level of 0.37) was used for binarization and the ‘bwarea’ function for the white pixel area calculation. Note that the obtained area has an arbitrary unit based on the calculation of the nearest pixels. The saw-cut area of the reference ($SA_{ref}$) was calculated as $4.5\times 10^{4}$. The computed $SA_{ref}$ value can be considered as the optimal saw-cut area in practice, considering that it does not exhibit raveling damage.

**Figure 9:**
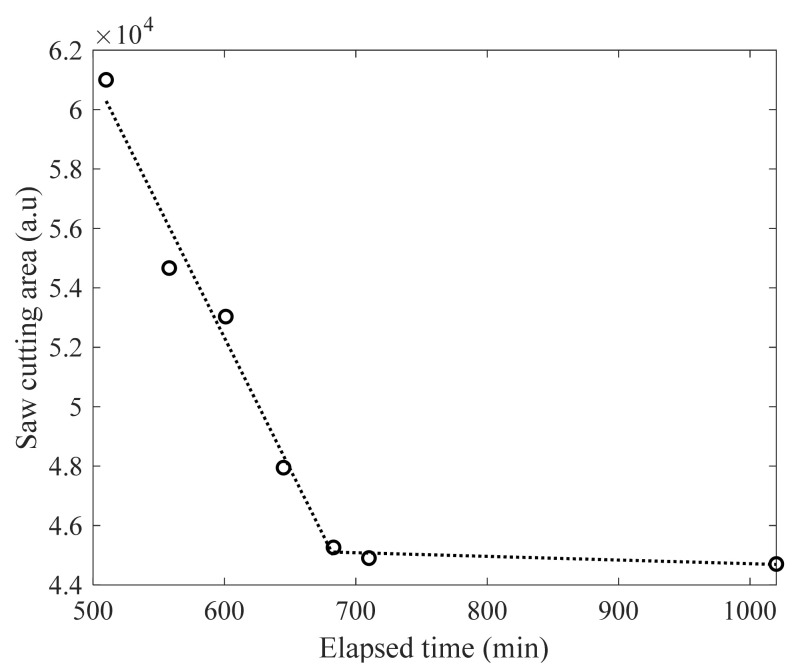
The obtained saw-cut areas indicating two distinct regions showing different slopes.

The quantified saw-cut areas as a function of elapsed time are presented in Figure 9. As the saw-cut introduction time increases, the saw-cut area computed by the image processing scheme decreases and converges to a certain level of the saw-cut area. Compared to the final saw-cut area, the raveling damage was identified implying that the saw cut quality is highly time-dependent. The saw-cut areas seen in Figure 9 are divided into two regions, including the steep and gentle slopes of the trend lines (the slopes as −88.5 and −1.2, respectively). The inflection point is about 675 min, which is close to the setting time defined by leaky Rayleigh waves (650 min).

### 4.2. Optimal Saw-Cutting Time Based on Leaky Rayleigh Waves Velocity

**Figure 10:**
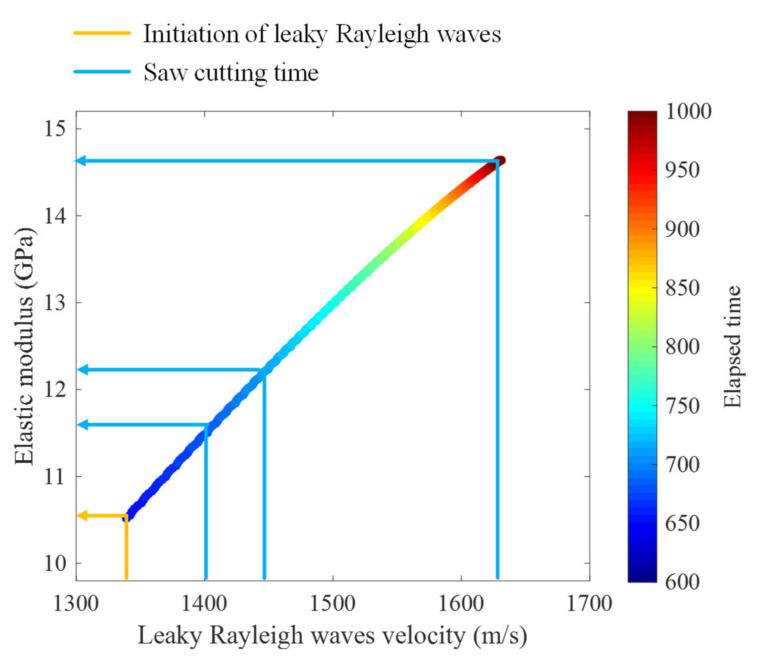
Elastic modulus of concrete computed using measured leaky Rayleigh wave responses. Elapsed time is presented in a colormap.

Based on the proposed signal processing scheme described in Section 2, the leaky Rayleigh wave velocity is converted to the elastic modulus of concrete. Given a leaky Rayleigh wave velocity obtained from experimental data, the corresponding elastic modulus is computed using Equation (7) which was derived from the numerical simulation results shown in Figure 2. In Figure 10, the relationship between the leaky Rayleigh wave velocity and elastic modulus is presented. The elapsed time after concrete hardening started is presented in a colormap. Four points including the initiation of leaky Rayleigh waves and three saw-cut introduction times are indicated in the figure. The elastic moduli at the four points are 10.5, 11.6, 12.2 and 14.6 GPa, respectively. The mechanical property would give a better understanding of the material behavior under dynamic loading such as a saw cut.

**Figure 11:**
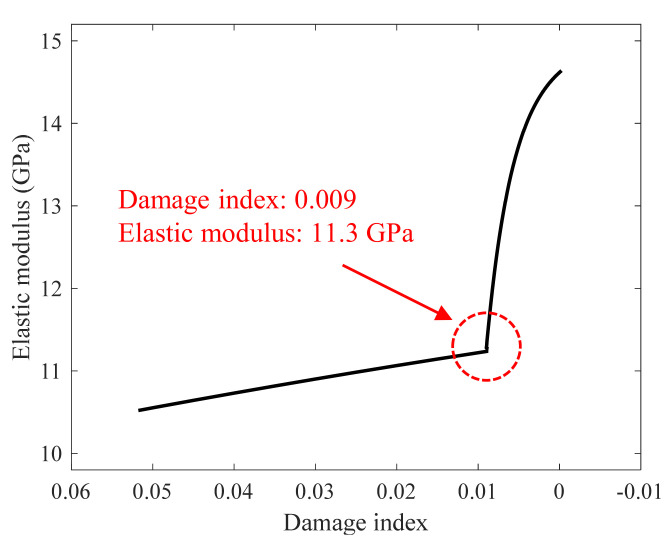
Correlation between the damage index of a saw-cut area and elastic modulus of concrete.

To evaluate the degree of raveling damage, a damage index (DI) is calculated based on the reference, given by

(9) $$DI=\frac{SA_{i}-SA_{ref}}{SA_{ref}},$$

where $SA_{i}$ is the saw-cut area at each trial. The damage index shown in Equation (9) indicates the relative ratio of the saw-cut area with respect to the optimal saw-cut area; *DI* values closer to zero represent the smaller extent of raveling damage. Figure 11 presents the correlation between the damage index and elastic modulus of concrete. Similar to the saw-cut area seen in Figure 9, the inflection point is observed at 11.3 GPa with a damage index of 0.009. After the inflection point, the damage index slowly converges to zero while the elastic modulus steeply increases. This implies that the optimal saw-cut initiation point can be considered the inflection point because the damage index at this point is less than 1%. Note that the initiation of leaky Rayleigh wave corresponds to the elastic modulus value of 10.5 GPa and damage index of 0.05. The presented results suggest that a saw cut can be introduced when the elastic modulus computed using the leaky Rayleigh wave velocity reaches approximately 11.5 GPa.

## 5. Conclusions

In this study, a wireless and contactless system was applied to evaluate the optimal saw-cutting time based on leaky Rayleigh wave measurements. The development of sensor networks and the proposed signal processing approach were numerically and experimentally validated. Based on the results, the following conclusions were drawn:

(1) The wireless and contactless ultrasonic system measures the initiation of leaky Rayleigh waves and their velocity increments during concrete hardening. The performance of the developed hardware was nearly identical to that of the wired system.
(2) The proposed signal processing scheme provides the velocity of leaky Rayleigh waves and the corresponding elastic modulus of concrete, which is a conservative prediction compared to theoretical Rayleigh wave velocity.
(3) Graphical analysis efficiently shows time-dependent raveling damage, and a damage index is calculated to assess the damage level with respect to the development of elastic modulus.
(4) The initiation of leaky Rayleigh wave with a 5° incident angle was observed two hours later than the final setting time defined by the pin penetration test, which represents 5% of raveling damage compared to the optimal saw-cutting time.
(5) The correlation between the damage index and elastic modulus implies that the saw cutting would be optimally performed when the elastic modulus of concrete reaches approximately 11.5 GPa.

## Figures and Tables

**Table 1:** Summary of the parameters used for numerical simulations.

Parameters			Value	Parameters		Value
Mesh properties	Type		Triangular mesh	Excitation source	Type	Sinusoidal wave (3 cycle)
	Size	Max	37.1 mm		Center frequency	50 kHz
		Min	0.21 mm	Concrete	Elastic modulus	0.1~25 GPa (1 GPa interval)
Model dimensions	Concrete		700 × 150 mm		Density	2300 kg/m^3^
	Air		700 × 150 mm		Poisson’s ratio	0.2
Total time duration per simulation			800 µs	Air	Density	1.23 kg/m^3^
Time step *			88~710 ns	Solver type		Time-Explicit

* A time step is automatically selected by COMSOL Multiphysics depending on material properties for a stable time stepping.

**Table 2:** Mixture design proportions.

Maximum Aggregate Size (mm)	Slump (mm)	Water to Cement Ratio	Volume of Entrained Air (%)	Unit Weight (kg/m^3^)					
				Water	Cement	Fine Aggregate	Coarse Aggregate	Fly Ash	Admixture (Superplasticizer)
25	40	0.47	7	167	353	709	1079	88	3

## Data Availability

The data presented in this study are available upon request from the corresponding author.
